# “Double-Hit” Chronic Lymphocytic Leukemia, Involving the *TP53* and *MYC* Genes

**DOI:** 10.3389/fonc.2021.826245

**Published:** 2022-01-13

**Authors:** Florence Nguyen-Khac

**Affiliations:** ^1^ Cell Death and Drug Resistance in Lymphoproliferative Disorders Team, Centre de Recherche des Cordeliers, INSERM UMRS 1138, Paris, France; ^2^ Sorbonne Université, Paris, France; ^3^ Service d’Hématologie Biologique, Hôpital Pitié-Salpêtrière, AP-HP, Paris, France

**Keywords:** TP53, MYC, 17p deletion, chronic lymphocytic leukemia, MYC gain, MYC translocation

## Abstract

Although the 17p deletion [del(17p)] is rare in cases of treatment-naive chronic lymphocytic leukemia (CLL), its frequency is higher in refractory/relapsed CLL – particularly in patients undergoing chemo(immuno)therapy. *TP53* disruption (deletion and/or mutation) is the strongest prognostic factor for refractoriness to chemotherapy; the use of Bruton tyrosine kinase inhibitors and BCL2 inhibitors is then indicated. Rare cases of CLL can also harbor translocation or gain of the *MYC* oncogene. “Double-hit CLL” (with del(17p) and *MYC* gain) is associated with a very poor prognosis. The prognostic impact of *TP53* disruption with *MYC* aberrations in patients receiving targeted therapies must now be evaluated.

## Introduction

Loss of the short arm of chromosome 17 [del(17p)] results from various chromosomal abnormalities, including deletions, translocations, isochromosomes, and ring chromosomes. All these chromosomal abnormalities lead to the loss of one copy of the *TP53* gene (located at 17p13) in patients with chronic lymphocytic leukemia (CLL), and the remaining allele is mutated in more than 90% of cases. del(17p) is often associated with a complex karyotype (three or more chromosomal abnormalities) (1). Rare CLL cases can also harbor translocation or gain of the *MYC* gene, independently or in association with del(17p) (1–3).

### *MYC* Translocation

The *MYC* oncogene (located at 8q24) is a transcription factor involved in many biological mechanisms, including as cell cycle control, apoptosis, cell growth, and cell differentiation. The translocation t(8;14)(q24;q32) and its variants t(8;22)(q24;q11) and t(2;8)(p11;q24) are typically associated with Burkitt lymphoma; *MYC* then comes under the control of an immunoglobulin heavy chain enhancer, a lambda light chain enhancer or a kappa light chain enhancer, respectively. *MYC* also has non-immunoglobulin gene partners. These translocations can be observed in other B cell neoplasms, such as diffuse large B-cell lymphoma (DLBCL), B-prolymphocytic leukemia (B-PLL) and CLL (4, 5). The World Health Organization’s classification of large B-cell lymphomas now includes a new entity called “double hit high-grade B cell lymphoma” (HGBL), in which *MYC* rearrangement is combined with a *BCL2* and/or *BCL6* rearrangement (6). This category of double- or triple-hit lymphomas only comprises translocations involving *MYC* and the two other genes; hence, lymphomas expressing *MYC* with *BCL2* and/or *BCL6* (according to immunochemical assessments) but that lack translocations are not encompassed by the definition (7).

### *MYC* and Transformed Indolent B Cell Malignancies

*MYC* is often involved in transformed indolent mature B neoplasms, such as the transformations of follicular lymphoma (FL) to DLBCL and CLL to Richter syndrome (8). Transformation of FL occurs in 25-35% of cases. A very small proportion of cases of FL (<0.5%) harbor a t(*MYC*), and a progression to a HGBL double hit may occur in cases with both t(14;18) and t(*MYC*) (6). Extra copies of *MYC* can also be observed in FL but (unlike t(*MYC*)) do not appear to be associated with a risk of transformation (9). Although *MYC* translocation/activation is rare in FL, up to 75% of cases of transformed FL show a gain in MYC activity (8). With regard to DLBCL-type Richter syndrome, the MYC pathway is deregulated in about 70% of cases, and somatic structural *MYC* alterations are present in 30% of cases. *MYC* deregulation is often acquired upon transformation (10).

### *del(17p)* and *MYC* Aberrations in B-Prolymphocytic Leukemia

*MYC* translocations (t(*MYC*)) are frequent in B-PLL (4). In a recent study, we found that 21 of the 34 cases (62%) of B-PLL had a t(*MYC)*. Furthermore, the translocated *MYC* gene was mutated in 3 of the 10 tested cases (30%). *MYC* gain was also observed in this disease, albeit at a lower frequency (5 out of 34, 15%) than t(*MYC*). Interestingly, t(*MYC*) and *MYC* gain were mutually exclusive; t(*MYC*) was present in the major clone, and *MYC* gain was mainly subclonal. It is noteworthy that *MYC* gain was associated with a highly complex karyotype, with five or more chromosomal abnormalities. We have shown that B-PLL patients with an MYC aberration (translocation or gain) and a del(17p) had the worse prognosis. In all evaluable del(17p) B-PLL cases, the remaining *TP53* allele was mutated. However, the small sample size prevented a statistical analysis of *TP53* mutational status and *MYC* aberration. Thus the combination of *MYC* and a *TP53* aberration is associated with a very high-risk form of B-PLL (4).

### del(17p) and *MYC* Aberrations in CLL

In contrast to B-PLL, translocations involving *MYC* are very infrequent (<0.5%) in CLL (2, 11). t(MYC) is often a secondary event in the course of the disease and is associated with a complex karyotype, an elevated prolymphocyte count, and an aggressive form of CLL (2). The *MYC* gene is also involved in 8q24 gain, which is detected in less than 0.5% or 3-4% of cases of CLL (using chromosome banding and microarrays analyses, respectively) (11–14). Gain of 8q can occur early in the course of CLL (15). It has been linked to a complex karyotype, a shorter overall survival time, and a shorter time to first treatment (11–14). Overall, *MYC* abnormalities – whether translocations or gains – are associated with a poor prognosis in CLL.

**Figure 1:**
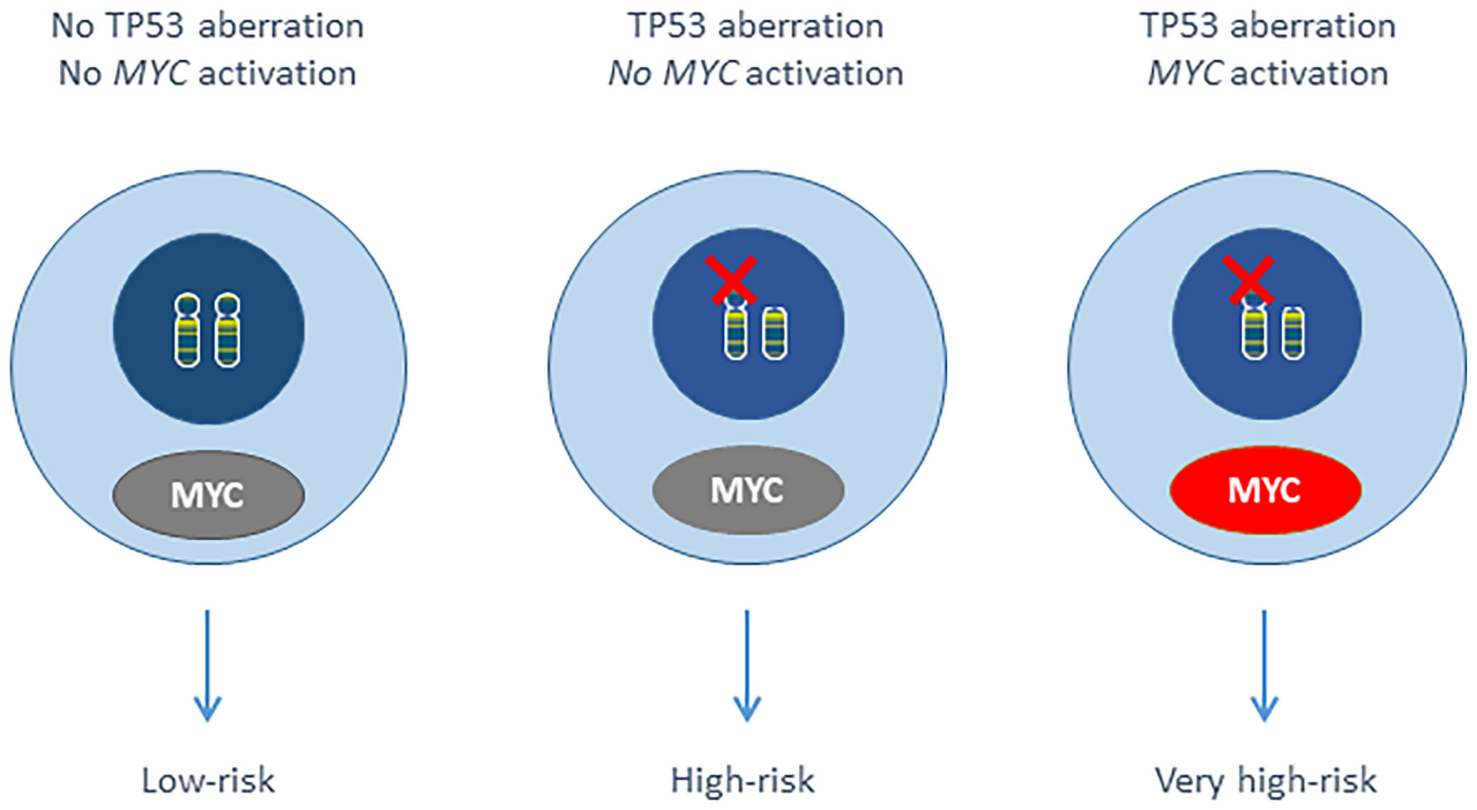
Putative scheme of double-hit CLL.

Harbel et al. showed that del(17p) occurred with a more than 3-fold increase in a cohort of 33 t(*MYC*) CLL compared to general CLL (3). The frequency of *MYC* gain is higher in CLL with del(17p) (ranging from 9% to 44%) (13, 14, 16–18), and we have demonstrated that the del(17p) + 8q24 gain combination (involving *TP53* and *MYC* respectively) was associated with a very poor outcome within the del(17p) CLL. The remaining *TP53* allele was mutated in 55 (92%) of the 60 evaluable del(17p) patients. The small number of cases prevented a statistical analysis of *TP53* mutational status and *MYC* gain. It should be noted that there were not t(*MYC*) cases in our del(17p) CLL series (n=195). By analogy with double-hit HGBL, we identified double-hit CLL as an aggressive form of the disease (**Figure 1**) (1, 6).

### Cooperation Between *MYC* and *TP53* Defects

It has been shown that *MYC* and *TP53* defects cooperate in MYC-induced murine lymphomas. In Eμ-MYC transgenic mice, MYC activation strongly selected for surviving cells, with inactivation of the ARF-Mdm2-p53 pathway (19). Thus lymphomagenesis in MYC mouse models requires additional genetic alterations - such as loss of p53 (20). In our del(17p) CLL series, *MYC* gain and del(17p) were in the same clone in 8 (62%) of the 13 evaluable cases, *MYC* was gained before the del(17p) in 3 cases (23%), and *MYC* was gained after the del(17p) in 2 cases (15%). It is noteworthy that 6 of the 13 (46%) cases carried the der(17)t(8;17) abnormality, with an unbalanced translocation between the short arm of chromosome 17 and the long arm of chromosome 8; this results in both *MYC* gain and del(17p) (1). Regarding t(*MYC*) in CLL, Put et al. described a case with t(*MYC*) before del(17p) and a case with t(*MYC*) and del(17p) in the same clone. In B-PLL, the majority of the 7 evaluable cases with t(*MYC*) and del(17p) in the literature had both abnormalities in the same clone (6/7); the last case had the del(17p) before t(*MYC*) (2, 4). There were two B-PLL cases with both *MYC* gain and del(17p): *MYC* gain and del(17p) were present in the same clone for one patient, and *MYC* was gained after del(17p) in the other patient (4). Overall, it is difficult to draw conclusions about the order of appearance of these two abnormalities, except in cases with the der(17)t(8;17). The two types of longitudinal event (*MYC* followed by *TP53* aberrations, and *TP53* followed by *MYC* aberrations) may exist.

### BCR Signaling

Given that TP53 downregulates BCR signaling, and MYC represses downregulators of BCR signaling, both *TP53* and *MYC* aberrations might results in elevated FOXP1 levels. One can reasonably hypothesize that a combination of an *MYC-*activating aberration (repressing miR-150 and miR-34a) and *TP53* deletion/mutation (further repressing miR-34a) can lead to very prominent activation of FOXP1 and then the BCR. Both miR-150 and miR-34a target FOXP1, albeit at different positions (21–24).

It would be interesting to evaluate the response to Bruton tyrosine kinase inhibitors (BTKi) in patients with double-hit CLL. As MYC acts as a key downstream BCR effector, its overexpression is known to rescue the absence of BCR activity in some B cells (8, 25). Indeed, upregulation of *MYC* has been observed in ibrutinib-resistant mantle cell lymphoma cell lines (26). Treating CLL with *TP53* and *MYC* aberrations might be challenging. Intriguingly, it has been shown that in a context of chemotherapy in B-cell lymphoma with inactive p53, MYC gain can be used to over-activate cells and induce apoptosis (27).

### “Double-Hit” CLL

The concept of a double hit involving the *MYC* gene in HGBL could be thus extended to other B cell malignancies in general and B-PLL and CLL in particular. When combined with del(17p) in B-PLL and CLL, *MYC* aberrations (translocations or gains) appeared to be associated with a very poor prognosis. However, only retrospective cohorts have been studied to date, and most patients were undergoing chemo(immuno)therapy. Moreover, *TP53* mutational status must be further evaluated, in order to confirm that the combination of a *TP53* mutation [and not only del(17p)] with a *MYC* aberration results in a poor prognosis. Given the low frequency of CLL cases with *MYC* aberrations, and the low proportion of cells with *MYC* aberrations (in case of a subclonal abnormality) and thus the requirement for systematic screening with a fluorescent *in situ* hybridization (FISH) probe, it will be challenging to evaluate the prognostic impact of these two abnormalities in prospective trials of targeted therapies (e.g. BTKi and BCl2 inhibitors). However, understand the mechanisms of resistance to new drugs is essential, and any aggressive abnormalities must be carefully analyzed. Although t(*MYC*) is easy to observe by karyotype, the *MYC* gain might be difficult to detect. In CLL, we recommend karyotyping and systematic FISH analysis with *TP53* and *MYC* probes prior to the initiation of each line of treatment. It is noteworthy that *MYC* and *TP53* aberrations can be present in a subclone and so might be overlooked by techniques like chromosomal microarrays, multiplex ligation-dependent probe amplification, massively parallel sequencing, and optical genome mapping. FISH is still the most sensitive technique for detecting chromosomal gains and losses. Of course, *TP53* mutation analyses should (in addition to FISH) be performed in CLL (28).

In conclusion, the results of a retrospective study showed that del(17p) and 8q gain (involving *TP53* and *MYC*, respectively) are associated with a very poor prognosis in CLL. This very high risk of double-hit CLL must now be confirmed (including the impact of *TP53* mutation status and rare translocations involving *MYC*) for the targeted therapies (e.g. BTKi and BCL2 inhibitors) now used as first-line treatments.

## Author Contributions

The author confirms being the sole contributor of this work and has approved it for publication.

## Conflict of Interest

The author declares that the research was conducted in the absence of any commercial or financial relationships that could be construed as a potential conflict of interest.

## Publisher’s Note

All claims expressed in this article are solely those of the authors and do not necessarily represent those of their affiliated organizations, or those of the publisher, the editors and the reviewers. Any product that may be evaluated in this article, or claim that may be made by its manufacturer, is not guaranteed or endorsed by the publisher.
